# *QuickStats:* Age-Adjusted Death Rates* from Unintentional Falls Among Adults Aged ≥65 Years,^^†^^ by Sex — National Vital Statistics System, United States, 2000–2015

**DOI:** 10.15585/mmwr.mm6635a6

**Published:** 2017-09-08

**Figure:**
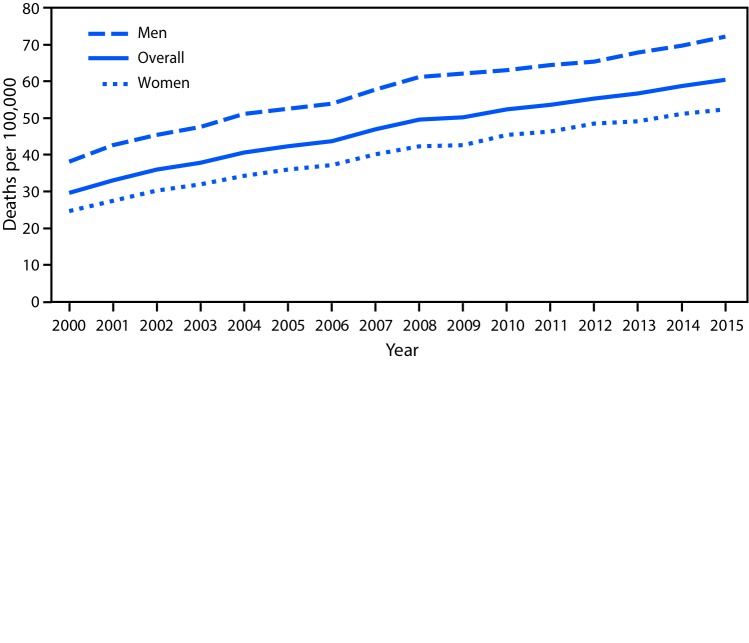
From 2000 to 2015, the age-adjusted unintentional fall death rate for adults aged ≥65 years increased an average of 4.9% per year. The death rate for women increased from 24.6 to 52.4 per 100,000 population. The death rate for men increased from 38.2 to 72.2. Throughout the period, men had higher death rates than women.

**Source:** National Vital Statistics System. Underlying cause of death data, 2000–2015. https://wonder.cdc.gov/ucd-icd10.html.

